# Development of Augmented-Reality-Based Magnetic Field Visualization System as an Educational Tool

**DOI:** 10.3390/s22208026

**Published:** 2022-10-20

**Authors:** Hisahide Nakamura, Yukio Mizuno

**Affiliations:** 1 R&D Division, TOENEC Corporation, Nagoya 457-0819, Aichi, Japan; 2 Department of Electrical and Mechanical Engineering, Nagoya Institute of Technology, Nagoya 466-8555, Aichi, Japan

**Keywords:** magnetic field, visualization, augmented reality, electrical equipment

## Abstract

Electromagnetism is a difficult subject to learn because the phenomenon is not observed directly and does not have a perceivable, concrete image. The visualization of the phenomenon will greatly help beginners understand electromagnetics. This paper proposes an augmented-reality-based visualization system of the magnetic flux density around current-generating objects. The system can be realized with a portable magnetic field sensor and a familiar device—a smartphone. The effectiveness of the system is verified through experiments, and our findings suggest that the system can effectively help an operator gain an intuitive understanding of the magnetic flux density.

## 1. Introduction

Electromagnetism is generally difficult for students to understand because of its abstractness. Some students lose their motivation to learn it early on, whereas some only manage to understand it vaguely. The invisibility of electric, magnetic, and electromagnetic fields makes it challenging for students to have specific or concrete images of these phenomena, resulting in insufficient understanding.

In a study on the visualization of the magnetic flux density, Nishimura et al. proposed a visualization system of the radial and vectored magnetic field distribution around a spherical surface based on magnetic computed tomography [1]. This system can visualize the magnetic flux density around a source, but measurement application is limited to the magnetic field of that specific source. The effect of magnetic fields should be visualized more freely (i.e., without restrictions in measurement range) to help beginners understand electromagnetics.

Augmented reality (AR) is a promising and powerful visualization tool. It can display computer-generated virtual images in the real world. With the emergence of high-performance computers, AR has been used in various fields, such as navigation systems and entertainment [2,3,4].

Several studies have been performed on the application of AR to help beginners understand electromagnetics. Matsumoto et al. proposed an AR-based real-time visualization system for electromagnetic education [5]. It can show the image of a magnetic field generated by a bar magnet with a web camera and a PC. Furthermore, they developed an immersive, real-time visualization system of 3D magnetic fields for educational purposes; the system is used with a head-mounted display [6]. It visualizes magnetic flux lines around permanent magnets by combining the magnetic flux density vector based on the Biot–Savart law and the magnetic moment method with the virtual particle method. Liu et al. proposed a magnetic field visualization method that can not only visualize magnetic field lines but also present the approximate sparse distribution of the magnetic field lines in space [7]. The magnetic flux density is calculated based on the Biot–Savart law with the Ampere molecular circulation hypothesis, and a particle tracking algorithm is adopted for real-time calculation. The magnetic flux lines around permanent magnets are visualized in 3D.

Authors have been investigating the visualization of low-frequency magnetic fields. In one system, the 3D magnetic field distribution is obtained by spot measurements in space in a short time with the aid of wireless transmission technology and a Kinect, a depth sensor developed by Microsoft [8]. In another work, this system was improved by expanding its frequency range and adding the FFT analysis function. Furthermore, with the use of AR, the magnetic fields in spot measurements in space were visualized in a real image with points of different colors denoting their strength [9]. As a Kinect sensor is used in both systems, the spot measurement area is limited to about 5 m from the Kinect sensor, and measurement cannot be performed when an object stands between the Kinect sensor and the magnetic field sensor. Moreover, the setup of a Kinect sensor is time-consuming.

Although several methods have been proposed for grasping electromagnetic profiles, it would be beneficial to visualize the levels of the magnetic flux density around a source with a familiar, portable device, such as a smartphone, based on a simple principle. With the progress of the performance of smartphones, some free or inexpensive applications have been developed for measuring the magnetic flux density using the sensors installed in smartphones. However, such applications perform poorly [10], according to a study where the power frequency magnetic flux density generated using a Helmholtz coil was measured with such applications and with a calibrated magnetic field meter. A reliable, external sensor is required when building visualization systems using smartphones.

Considering the above problems, a simple, portable visualization system of magnetic fields is proposed as an educational tool. It consists of an external probe for the magnetic flux density measurement, a magnetic field sensor for data processing, and a smartphone for display. The level of the magnetic flux density is displayed in real time on the smartphone using AR. For demonstration, this system is used to visualize the magnetic fields around an energized straight conductor, an electric heater, and an induction motor.

## 2. Visualization System of Magnetic Field

### 2.1. Structure of Sensing System

**Figure 1:**
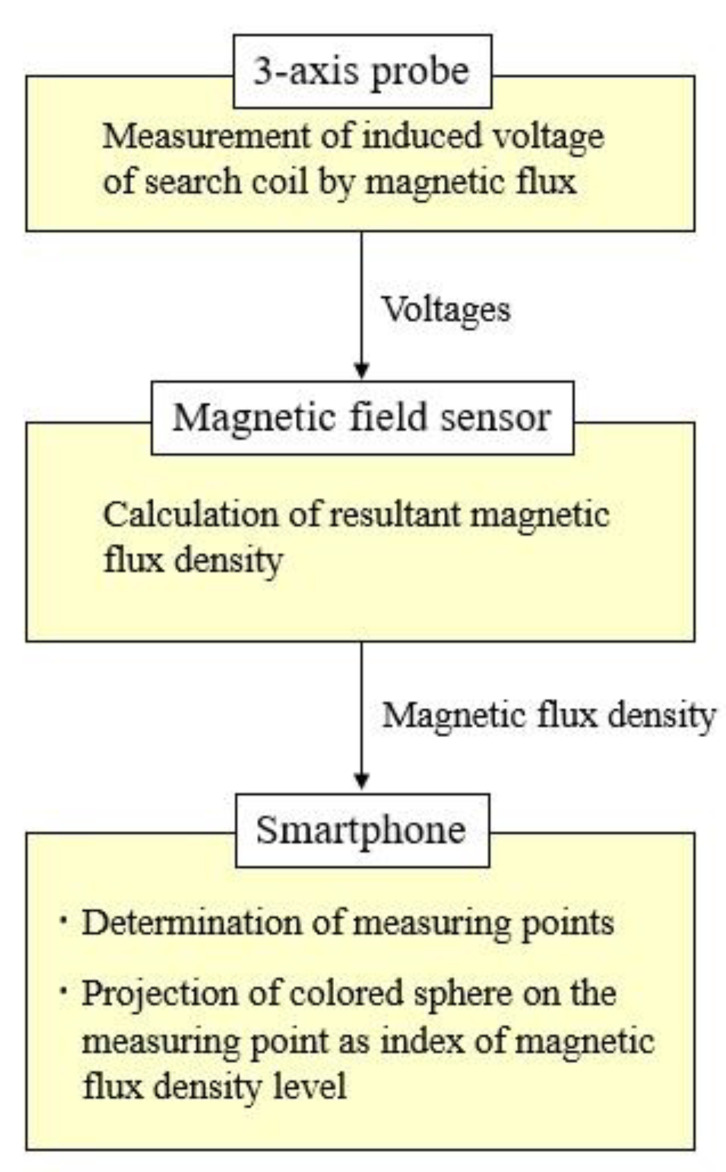
Configuration of visualization system.

The configuration of the visualization system, which consists of a three-axis probe, a magnetic field sensor, and a smartphone, is shown in Figure 1. The output of the probe is transferred to the magnetic field sensor, and the resultant magnetic flux density is calculated. In the smartphone, the measurement points of the magnetic flux density are confirmed. Then, a colored sphere indicating the level of the magnetic field density is projected on each measurement point in the image appearing on the smartphone display.

### 2.2. Magnetic Field Sensor

A lightweight, portable, easy-to-handle magnetic field sensor was developed for the rapid, easy measurement of the magnetic flux density in space. The magnetic field sensor is sized 80 mm (width) × 34 mm (height) × 145 mm (depth) and weighs about 210 g. The magnetic field sensor is handy and can be moved freely in the measurement space.

The three-axis probe for measuring the magnetic flux density is a 1.5 cm cube that is connected to the magnetic field sensor. The voltages induced by an AC magnetic field are measured with three concentric coils wound around the three axes. The magnetic field sensor has full-scale values of 25 and 125 μT, which can be switched using the sensor buttons. The frequency range of the magnetic sensor module is 40–1000 Hz, and the sensor resolution is 0.2 μT.

The magnetic field sensor receives output voltages from the three-axis probe and calculates the magnetic flux density of each axis and the resultant magnetic flux density. Then, the resultant magnetic flux density is stored. The sensor module is calibrated using a Helmholtz coil. The error of this sensor is less than ±5%. The resultant magnetic flux density calculated by the magnetic field sensor is transferred serially to the smartphone at 0.3 s intervals via a USB cable.

### 2.3. Data Processing with a Smartphone

Measurement points are positioned in the visualization system using ARCore, which is included in smartphones running on the Android operating system [11]. The ARCore tool kit is Google’s platform for building AR environments. It can estimate the position of a smartphone in real time by analyzing the images captured with a smartphone camera and the data from sensors detecting movement, such as angular speed and acceleration.

First, the camera position is acquired based on the starting position of the smartphone movement. Next, the position of the magnetic field probe, which is located 30 cm away from the camera, is calculated. This procedure is realized with ARCore.

Additionally, a sphere is displayed at the position of the measurement spot in the image appearing on the smartphone screen. The color of the sphere reflects the magnitude of the measured magnetic flux density (between 0 and 25/125 μT). Dark blue is assigned to 0 μT, and the color changes with increasing magnitude to blue, green, yellow, orange, and red (for the maximum value). The value of the magnetic flux density is indicated numerically on the upper left portion of the smartphone screen.

**Figure 2:**
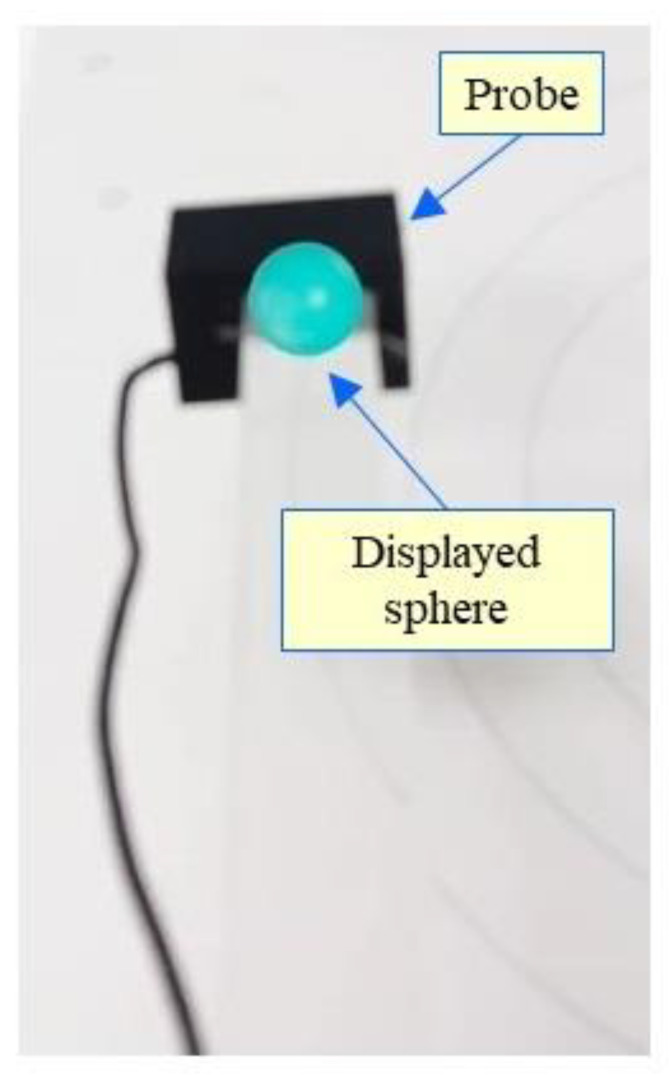
Image displayed on smartphone.

Figure 2 shows the displayed image on a smartphone during a spot measurement. The actual measurement point is the center of the sphere. The color of the sphere is light blue, which reflects a magnetic flux density of about 8.0 μT.

When the magnetic field is successively measured at another spot by moving the probe, a new sphere is displayed, but the previously displayed spheres remain on the screen until the end of the measurement. Thus, the number of colored spheres on the smartphone screen increases as the measurement proceeds. In this way, the visualization of the magnetic field is performed in the space of measurement. This series of visualized magnetic fields are simultaneously recorded as video images in the smartphone. Furthermore, the numerical data of the coordinates of the probe position and the measured magnetic flux density are linked and stored in a text file at 0.3 s intervals. Thus, the spatial distribution of the magnetic flux density can be determined by using a free 3D software program after measurement.

**Figure 3:**
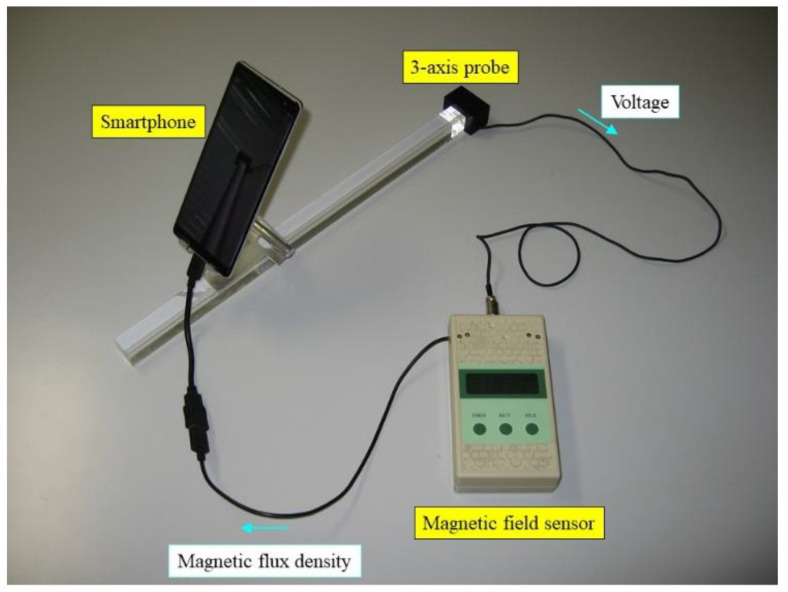
Proposed augmented reality (AR)-based visualization system.

**Figure 4:**
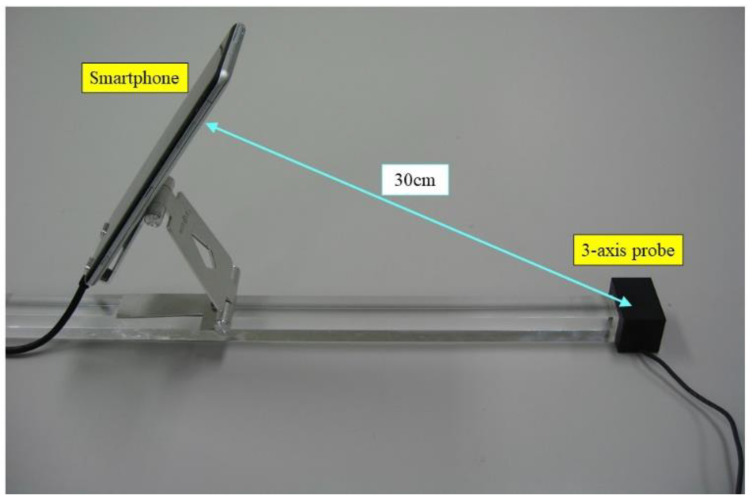
Distance between smartphone and probe.

Figure 3 shows the developed system, which enables the visualization of magnetic fields. One end of the acrylic rod is attached to the three-axis probe, and the other end is held by the operator. Then, the smartphone is affixed to the acrylic rod to maintain a constant distance between the smartphone camera and the probe. An arbitrary distance can be selected between the camera and the probe by changing the position of the smartphone along the rod. Here, the distance is fixed to 30 cm in consideration of operability, as shown in Figure 4.

## 3. Basic Application of Proposed System

### 3.1. Visualization of Magnetic Field

**Figure 5:**
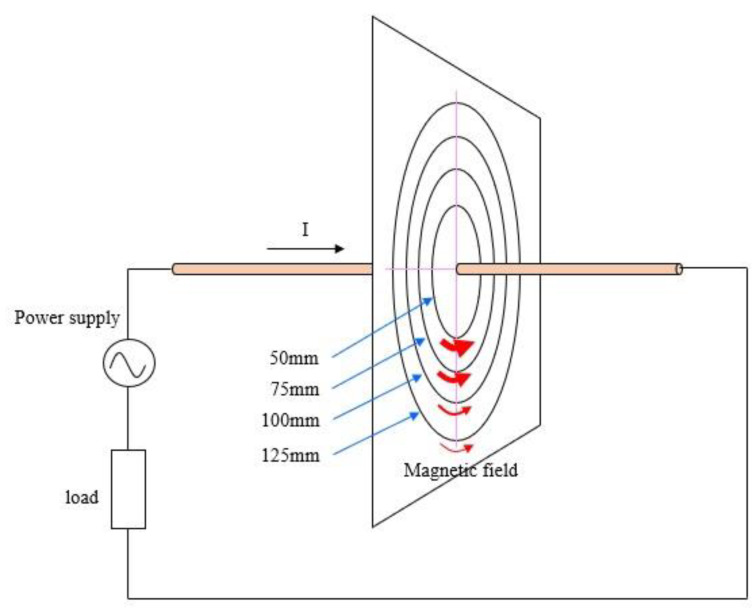
Experimental setup.

For a basic application example, the magnetic flux density around an electric current was visualized. Figure 5 shows the experimental setup. A flexible 100 V–15 A AC power supply code was used as the conductor, which was inserted into a rigid plastic pipe with an outer diameter of 6 mm to make a straight current path of about 6 m in length. The pipe was placed horizontally in space and pierced a 5 mm thick acrylic board perpendicularly. Four concentric circles with radii of 125, 100, 75, and 50 mm were drawn on the board. Attention was paid to avoiding the effect of other sources and objects. The distance between the conductor and the floor was set at about 800 mm, and the wiring connected to the conductor was placed far from it.

An AC current of 3.0 A at 60 Hz was supplied to the conductor to generate the magnetic flux density around the conductor. The magnetic flux density was measured by moving the probe along the circumference of the circles drawn on the board. The full-scale value of the sensor was set to 25 μT.

**Figure 6:**
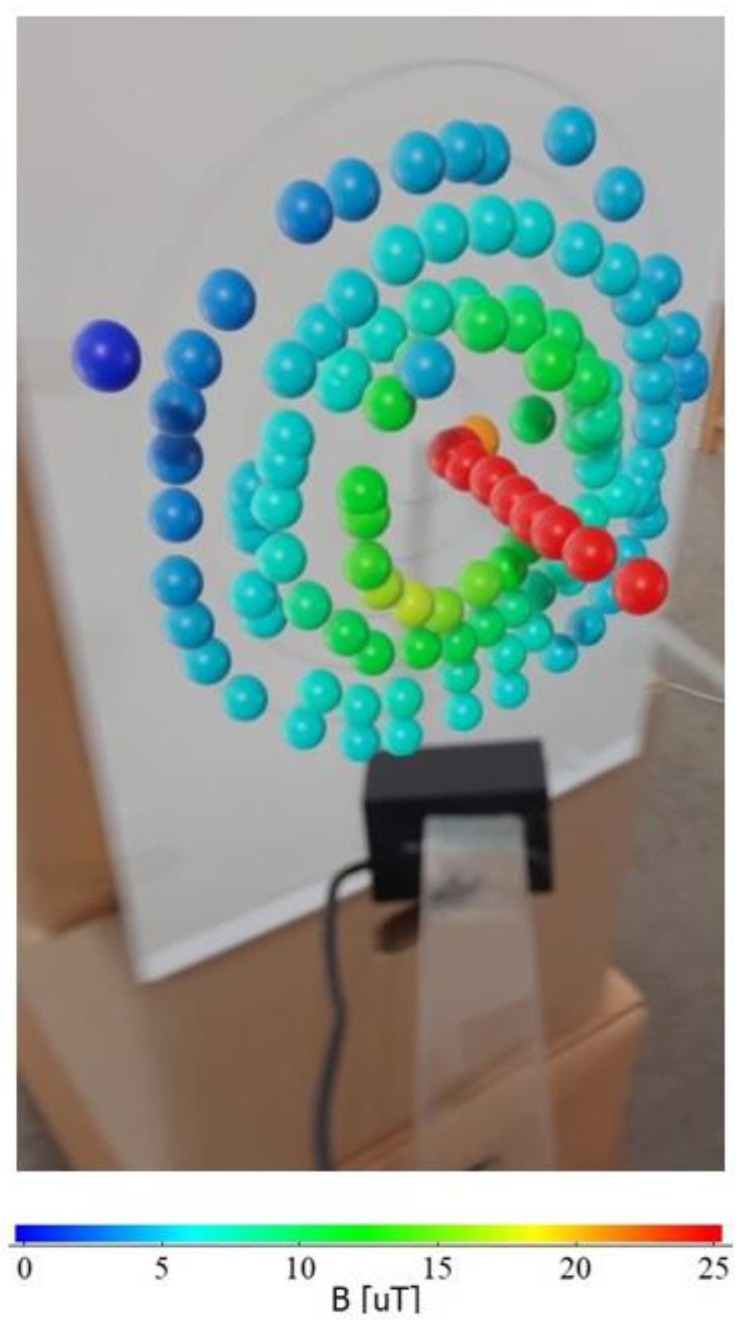
Visualized magnetic field generated using a linear conductor.

The visualized magnetic flux density, which increased as the measurement point approached the conductor, is shown in Figure 6. It can be confirmed that the magnetic field had almost the same color at the same radius. As the result, we can easily and visually understand the meaning of Ampere’s law.

### 3.2. Evaluation of Measured Value

Table 1 shows the average magnetic flux density measured along the circumference of each circle. The theoretical values, which were calculated by using Ampere’s law, are also shown in the table.

(1) $$B=\frac{\mu }{2\pi r}I$$

where an infinitely long straight conductor is assumed. The average magnetic flux density seemed acceptable for the purpose of the present study, and the visualization system was deemed suitable for evaluating the magnetic flux density in space.

The red spheres in Figure 6 show the magnetic field levels along the conductor. The distance between the conductor and the center of the probe was 1.5 mm, although there was a slight variation in the time of measurement. Red means a magnetic flux density of 25 μT or above in this case since the maximum value was set to 25 μT. The average measured magnetic flux density was 36.7 μT, as shown in Table 1. These results are reasonable, as the calculated magnetic flux densities at the distances of 15 and 20 mm from the conductor were 40 and 30 μT, respectively.

The proposed system visualizes the magnetic flux density on a smartphone display at the time of measurement by showing a numerical value and a sphere with a color that reflects the level of the magnetic field density. From an analysis of the recorded data, further details and accurate distribution of the magnetic field density are provided after the measurement. This system is expected to help beginners understand the magnetic flux density intuitively.

### 3.3. Evaluation of Probe Track and Positioning

In the proposed system, the probe position at a measurement point was determined by its 3D coordinates using the positioning function originally installed in a smartphone. Its validity is evaluated in this section.

The measurement of the magnetic flux density began at an arbitrary point in space. The probe was moved toward a display placed about 1 m from the starting point, and measurement was performed around the display. Then, the probe was visually moved back to the starting point to finish the measurement. The recorded coordinates of the starting point were compared with those of the finishing point. This procedure was conducted three times.

**Figure 7:**
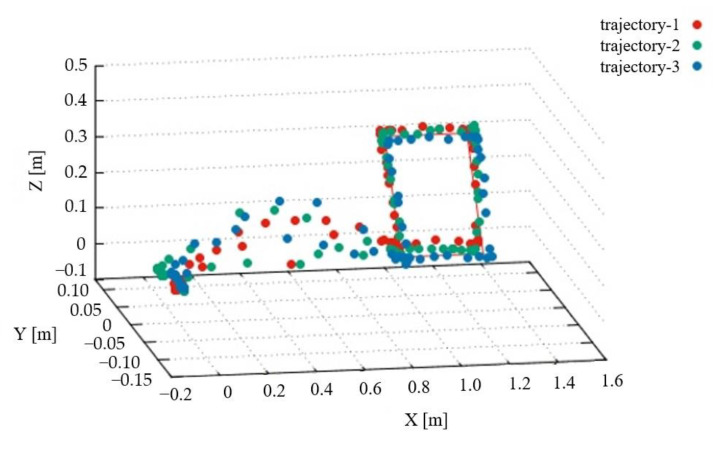
Trajectory of probe.

The probe tracks during the abovementioned procedure are shown in Figure 7, where the origin was set to the position of the starting point of the measurement. The red solid lines in Figure 7 denote the outline of the display. The tracks confirm that the probe moves according to the intention of an operator. Table 2 summarizes the discrepancies in the coordinates between the starting and finishing points in the three trials. Our findings show that the coordinates of the starting and finishing points were almost the same in the first and third trials. In the second trial, a difference of 43 mm was recorded in the y coordinate. Considering the principle and specifications of ARCore, the difference lies in the allowable range. This result is acceptable because it was obtained after moving the system by more than 1 m along the track.

## 4. Visualization of Magnetic Field around Electrical Equipment

Here, the proposed visualization system is applied to evaluate the magnetic flux density around electrical equipment. Field calculation around electric apparatus is generally laborious because of the complicated structures of such devices. The magnetic fields around an electric heater and an induction motor are visualized here, and the validity of the proposed system is discussed.

### 4.1. Electric Heater

**Figure 8:**
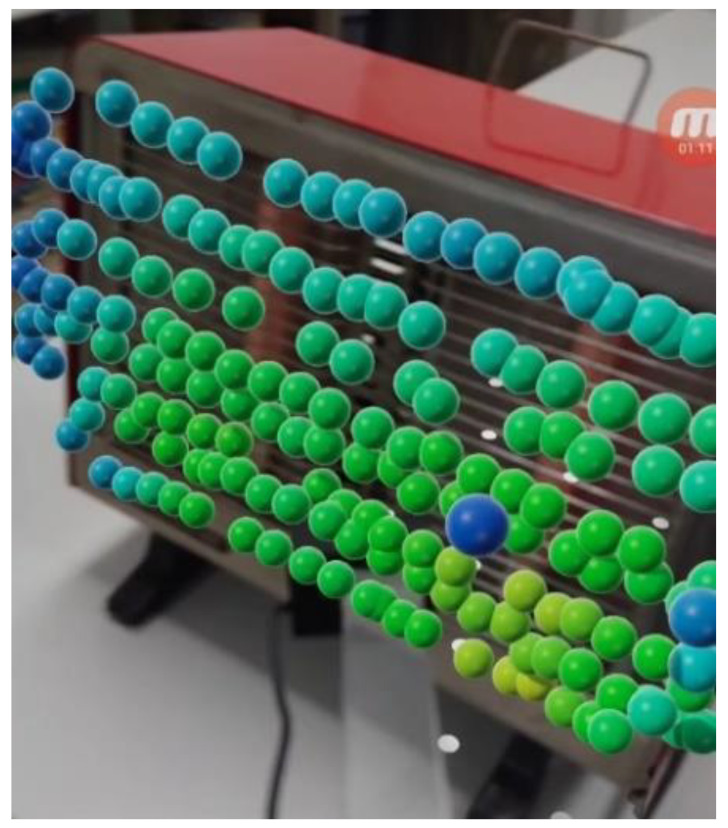
The visualized magnetic field generated by a heater.

An 800 W electric heater was selected as an object of magnetic field measurement. The measurement range of the magnetic field sensor was set to 25 μT, and the measurement was performed at more than 700 points around the heater. Figure 8 shows the smartphone display during the measurement, in which the colored spheres lie at the measurement points in front of the electric heater. Dark blue spheres indicate magnetic flux densities below 0.2 μT. On the other hand, as shown in Figure 8, some small white circles are displayed automatically when we operate AR Core. However, these circles do not relate to the measurement points.

**Figure 9:**
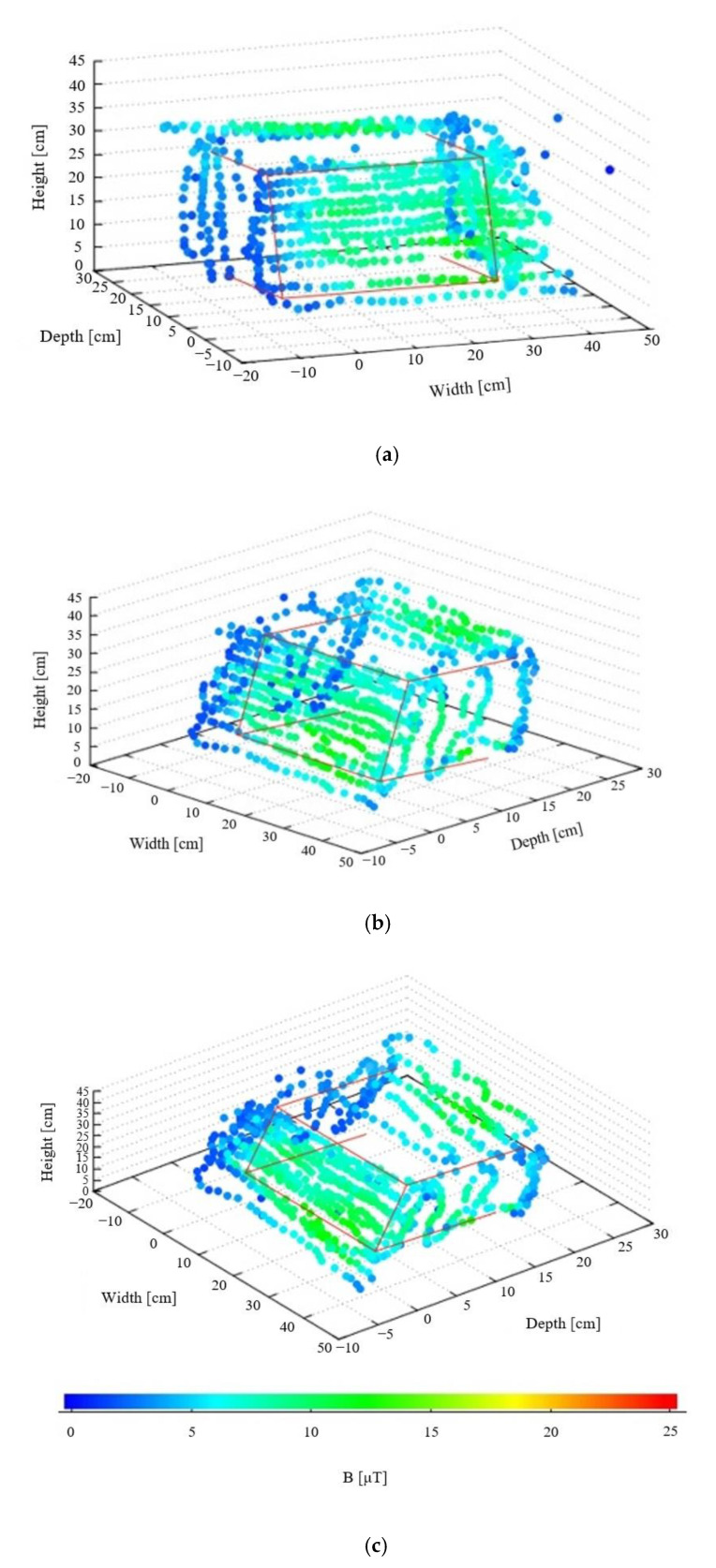
Visualized magnetic field around heater: (**a**) front view, (**b**) side view, and (**c**) top view.

The recorded data were analyzed after the measurement to further clarify the difference in the magnetic field. The magnetic field profiles around the heater can be visualized from three different angles using the coordinates and values of the magnetic field at more than 700 measurement points, as shown in Figure 9a–c. Only the data obtained at a distance of about 3 cm from the heater were used in these figures to avoid the superposition of the spheres. The origin of the coordinates was the bottom-left corner of the electric heater. The red lines outline the heater. From these figures, the location of the highest magnetic field is recognized at a glance. The maximum value was 17.4 μT.

### 4.2. Induction Motor

**Figure 10:**
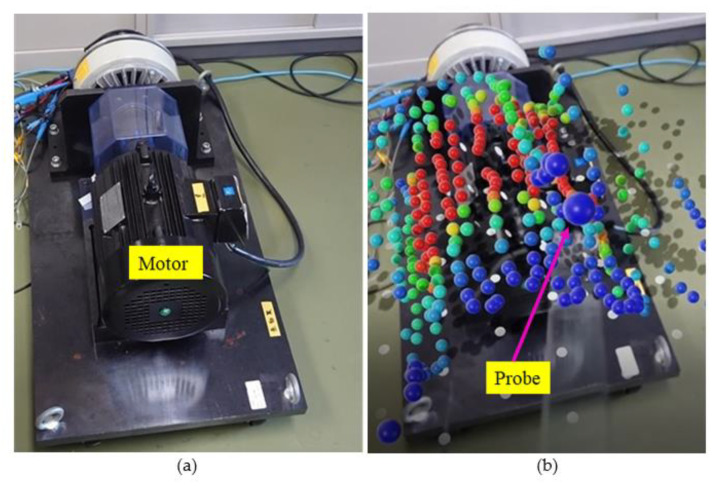
Visualized magnetic field around induction motor: (**a**) appearance of induction motor and (**b**) 3D distribution of magnetic field.

The magnetic flux density generated by another electrical equipment, an induction motor, was visualized, and the result is shown in Figure 10. The range of the magnetic field sensor was 25 μT. This figure confirms that the magnetic field was large outside the region where stator winding existed inside the motor. The change in the maximum range of the sensor reveals that the magnetic flux density exceeded 100 μT at some measurement points close to the housing surface. However, the magnetic flux density decreased with the distance from the motor.

It is difficult to calculate the magnetic flux density distribution around the motor theoretically. The quantification and visualization of the magnetic flux density were easily achieved using the proposed system, which verifies its usefulness.

### 4.3. Further Application

The adoption of the present system is not limited to educational objectives. It may be used in other valuable practical applications, such as the assessment of compliance with mandated values of the magnetic flux density. Depending on magnetic field sources and environments, several international standards and directives have been established to prescribe measurement procedures and/or to comply with threshold values [12,13,14]. These prescribed procedures are sometimes complicated and take time to implement. Moreover, a magnetic field should be measured in a short time because magnetic fields change depending on the time variations in currents.

The development of a reliable, easy-to-handle meter for measuring magnetic fields around sources in a short time would be useful, and the proposed system satisfies this requirement. It will be beneficial to conduct a rough assessment of magnetic fields before measurement in accordance with standards/directives and to obtain the 3D distribution of invisible magnetic fields around sources of interest. Measurement and evaluation will be performed as a future activity.

In the present study, the display method of an image on a smartphone screen was determined so that magnetic field levels and their distribution can be intuitively and qualitatively understood by those students who are beginning to learn about electromagnetism. Depending on the field source and/or the purpose of measurement, the display method of the image will be modified by changes in the program. For example, a more detailed color scale for spheres is helpful for understanding the magnetic field more quantitatively. A vectorial magnetic field can be displayed when the magnitudes of the magnetic field are stored in the direction of the three orthogonal axes.

## 5. Conclusions

In this paper, we proposed an AR-based visualization system of magnetic fields. The system was realized with a probe, a magnetic field sensor, and a familiar device—a smartphone. The output of the probe was transferred to the magnetic field sensor, and the resultant magnetic flux density was calculated. The measurement points of the magnetic flux density were confirmed in the smartphone. Then, a colored sphere indicating the level of the magnetic field density was projected on the measurement point in the image appearing on the smartphone display. The numerical value of the magnetic flux density was simultaneously specified on the smartphone display.

The magnetic flux density around an electric current was visualized to demonstrate the primary application purpose of this system as an educational tool. The magnetic flux density was measured by moving the probe along the circumference of concentric circles drawn on a board pierced by the conductor. Our findings confirmed that the magnetic flux density increased as the measurement point approached the conductor, almost coinciding with the theoretical values. This verified the ability of the system to aid students in understanding Ampere’s law.

Then, electrical equipment was targeted. The magnetic flux densities around an electric heater and an induction motor were visualized by means of colored spheres mapped on real images. The results were the same as the empirical prediction. Field calculation around electric apparatus is generally challenging because of their complicated structures, but the proposed system is useful in gaining an intuitive understanding of the magnetic flux density in such cases.

The miniaturization of the magnetic field sensor and the integration of the sensor and the rod holder are the required future tasks for improved system operability.

## Figures and Tables

**Table 1:** Measured magnetic flux density.

Radius of Circles (mm)	Magnetic Flux Density (μT)	
	Theoretical Value	Measured Value (Average)
125	4.8	4.7
100	6.0	6.1
75	8.0	8.1
50	12.0	11.9
15	40.0	36.7

**Table 2:** Error of probe position after measurement.

Trial	Error (mm)		
	*X* Axis	*Y* Axis	*Z* Axis
1	19	2	13
2	22	−43	11
3	6	−2	1

## Data Availability

Not applicable.
